# Suicide Risk Factors in Iranian Patients With Bipolar Disorder: A 21- Month Follow-Up From BDPF Study

**Published:** 2013

**Authors:** Amir Shabani, Samaneh Teimurinejad, Sadaf Kokar, Masoud Ahmadzad Asl, Behnam Shariati, Zohreh Mousavi Behbahani, Mohammad Reza Ghasemzadeh, Sahar Hasani, Mojgan Taban, Shahab Shirekhoda, Zahra Ghorbani, Somayeh Tat, Shabnam Nohesara, Seyed Vahid Shariat

**Affiliations:** 1 Mental Health Research Center, Bipolar Disorders Research Group, Iran University of Medical Sciences, Tehran, Iran.; 2 Tehran Psychiatric Institute, Iran University of Medical Sciences, Tehran, Iran.; 3 Mental Health Research Center, Iran University of Medical Sciences, Tehran, Iran.; 4 Tehran Psychiatry Institute, Iran University of Medical Sciences, Tehran, Iran.

**Keywords:** Bipolar Disorder, Cohort, Risk Factor, Suicide

## Abstract

**Objective**: Bipolar disorder is strongly associated with suicidal ideations, attempts and commissions. Although several studies have been conducted on suicide risk factors in patients with bipolar disorder worldwide, a comprehensive study has not been reported from Iran.

**Methods:** Patients with bipolar disorder type I, hospitalized in Iran Hospital of Psychiatry since May 2008 to August 2011 were sequentially enrolled. Patients were evaluated using Demographic and Clinical Variables Questionnaire, the Structured Clinical Interview for DSM-IV axis I disorders (SCID-I), Young-Mania Rating Scale (Y-MRS), and Hamilton Depressive Rating Scale-7 (HDRS-7). One hundred patients were followed for 2 to 42 months (mean: 20.6 ± 12.5 months).

**Results:** Only one patient attempted suicide during the follow-up period. 33% of the patients had history of previous suicide attempts. Female gender, divorce, and early age at onset of the disease were independently correlated with suicide attempt.

**Conclusion:** Suicide attempts rarely occur during systematic follow-up of patients with bipolar disorder type I. Larger samples and longer follow-ups are needed to confirm this finding.

**Declaration of Interest:** None.

## Introduction

In a systematic review of studies performed in Iran, Ghoreishi et al. found that the prevalence of suicide in general population of Iran was 9.4 in 100000c (1).Bokhara and colleagues in an epidemiologic sampling (Karaj) found that 12 in 100000 attempt to suicide in 10 months, leading to death in 0.2% of them (2).

Bipolar disorder is one of the leading psychiatric disorders related to suicide. According to epidemiologic researches, 29% of patients with bipolar disorder attempt to suicide at least once in their lifetime (3). In some studies conducted by health centers, 25-56 % of patients with bipolar disorder attempted to suicide at least once in their lifetime, causing death in 10-19 % of them (4,5).On the other hand, the lifelong prevalence of suicide attempts in patients with Bipolar type I disorder (BID) has been reported to be 10-18 % in another study (6).

Several studies on risk factors of suicide in bipolar patients conducted in other countries have revealed that factors including history of suicide attempt (7,8), psychiatric disorders in axis I or II (9), personality traits such as aggressiveness and impulsivity (9,10), severity of psychiatric disorders (11), age of onset of the psychiatric disorders (11-14), numbers of psychiatric hospitalizations (9,13), alcohol or substance use (11,12), suicidal thoughts (15), axis I co morbidities such as anxiety disorders (9,16), axis II co morbidities such as personality disorders (9) and history of childhood sexual or physical abuse (9), can increase the incidence of suicide attempts in these patients. Studies performed in Iran have focused on suicidal behaviors and their risk factors (17), prevalence of suicide attempts in clients of health centers (2), and predictive factors of suicide attempts in general population (18). To the best of our knowledge, comprehensive studies on suicidal behaviors of bipolar disorder patients are lacking in Iran.

We aimed to evaluate the occurrence of suicide attempt and its influential factors in BID patients by assessment of follow-up results and patients` history. 

## Materials and Methods

***Setting***

This was a prospective, naturalistic cohort study, part of a research entitled “The Bipolar Disorder Patients Follow-up (BDPF) project” granted by the Mental Health Research Center which its methods are published elsewhere (19).This study was performed on BID patients hospitalized in Iran Hospital of Psychiatry, Tehran, Iran.

*Patients*

One hundred BID patients, hospitalized in Iran Hospital of Psychiatry since May 2008 to August 2011, were recruited and followed up to 2 to 42 months (mean: 20.6 ± 12.5 months, median: 18.6 months) until October 2011.

Inclusion criteria were having BID diagnosed by a psychiatric attendant according to DSM-IV criteria (Diagnostic and Statistical Manual of Mental Disorders 4th Edition, Text Revision) and confirmed by a trained psychiatric resident according to Structured Clinical Interview for DSM-IV axis I disorders (SCID-I) , aged above 18, speaking Persian, permanent residency in Tehran, Karaj and their suburbs, having at least one landline number and a cell phone number, absence of mental retardation, and taking written informed consent.

The patients were evaluated at months 0, 2, 6 and every 6 months thereafter. In case of lack of patient cooperate for regular visits at the hospital for any reason, researchers visited the patient at his/her home, and if it was not possible either, phone call was utilized. Ultimately, if the patient was anyhow unreachable, he/she was dropped out.

*Main measurements*

The following instruments were utilized for information gathering and evaluations: Demographic and Clinical Variables

*Questionnaire* *:*

This Questionnaire includes questions about patient’s age, sex, marital status, education, accommodation, occupation, polarity of the mood episode at the disorder onset and enrollment in the study, axis I comorbidities, history of suicide attempts in first degree relatives, suicidal thoughts at the time of enrollment, number of prior hospitalizations during depressive, manic and mixed episodes.

The Structured Clinical Interview for DSM-IV axis I disorders (SCID-I):

SCID-I is a semi-structured interview for diagnosis of axis I disorders of DSM-IV (20). The Persian clinician version of the SCID-I has favorable reliability and validity (21).

*Hamilton Depressive Rating Scale-7 (HDRS-7):*

HDRS-7 has extensively been used in psychiatric researches for evaluating severity of depressive symptoms, with favorable reliability and validity even in telephone interviews (22).Mcintyre et al. reported that the 7-item HDRS had the same effectiveness as the 17-item one. Overall, the score of HDRS-7 ranges between 0-26 (23). The inter-rater reliability of HDRS among five interviewers of the present study was between 92-96% (19).

*Young-Mania Rating Scale (Y-MRS):*

Y-MRS is a clinical scale for evaluating the severity of manic symptoms and includes 11 items with total scores ranging 0 to 60 (24). The Persian version also showed good reliability and validity (25). The inter-rater reliability of Y-MRS among five interviewers of the present study was between 90-98% (19).

***Analysis***

Considering that only one patient attempted to suicide during the follow up period, the patients were retrospectively divided to two groups of with and without history of suicide attempts, prior to enrollment and then the variables in these two groups were compared.

We used SPSS-16 (Statistical Package for Social Sciences-16) for windows (SPSS Inc., Chicago, Ill.) for data analysis. Repeated measures analysis was used to determine the relationship between variables during study and suicide attempt. 

***Ethical issues***

The following ethical issues were considered in this study:

•All personal information was completely confidential. 

•Patients were not charged for the visits. 

•If the patient had serious suicidal thoughts or any emergent psychiatric problems, the researcher performed required interventions. 

•Informed consent was signed by the patient. 

•Patients were free to follow the study or not, whenever they wanted.

## Results

*Demographic findings*

Thirty three (33%) out of 100 patients were female and 67 (67%) were male with mean age of 35.9 (± 10.7) years. 37 (37%) were single, 47(47%) married and 16 (16%) divorced. 72 (72%) lived in their personal residential houses and 28 (28%) in rentals. Forty (40%) were unemployed, 4 (4%) were retired, 18 (18%) were housewives and 38 were (38%) employed. 65 (65%) had school education levels lower than high school diploma and 35 (35%) had high school diploma or higher.

*Clinical findings*

Patient’s first mood episode was major depressive in 20 (20%), mixed in 2 (2%), and manic in 78 (78%) patients. At the time of enrollment, 90 (90%), 7 (7%) and 3 (3%) patients were in manic, major depressive, and mixed episodes, respectively. At the first visit, 30 (30%) patients had axis I comorbidities including anxiety disorders in 11(11%) and substance use disorders in 19 ones.

Mean number of prior hospitalizations was 3.5. History of suicide attempt was positive in 33 (33%) and negative in 67 individuals (67%).

Overall, patients’ mean score was 26.5 ± 11.3 in YMRS and 3.5 ± 3.1 in HDRS. 6 (6%) had positive family history of suicide attempts in their first degree relatives. Five patients (5%) had suicidal thoughts at the time of enrollment according to HDRS-7.

Retrospective suicide attempt-related factors

The groups were compared considering gender, age, marital status, education, accommodation, occupation, polarity of the mood episode at disorder onset and enrollment in the study, axis I comorbidities, history of suicide attempts in first degree relatives, suicidal thoughts at the time of enrollment, number of prior hospitalizations during depressive, manic and mixed episodes, and mean scores in YMRS and HDRS at enrollment (Table 1).

Female gender, early age at onset of the disorder, divorce, educational level of diploma or higher, as well as family history of suicide attempts had significant relationship with history of suicide attempts. Results of multivariable logistic regression for evaluating independent relationship of the variables revealed that female gender, early age at onset of the disorder and divorce had independent relationship with history of suicide (Table 2).

*Follow-up findings*

During the follow-up period, 2 patients were dropped out of the study after the second interview and 9 ones after 18 months; including 2 cases of death due to myocardial infarction. 

Considering the average follow-up estimation, during the 21 months of follow-up only one person attempted suicide (0.57% in one year;95% confidence interval:0.52-.62%).

The only suicide attempter was a 55-year old married matron with elementary education and history of four previous suicide attempts. She had lost her sister as a result of suicide. The disorder onset was at the age of 36 with depression. She had suicidal thoughts at the time of admission and her mean scores in YMRS and HDRS-7 were 4 and 10 respectively.

**Table 1:** Comparing variables between two groups: bipolar I disorder patients (N=100) with and without history of suicide attempt

**Variables**	**With attempt history (n=33)**	**Without attempt history (n=67)**	**P value**
**Age, mean ± SD**	33.4 ±10	37 ±11	0.092
**Sex, N (%)** Female Male	17 (51.5) 16 (23.9)	16 (48.5) 51 (67.1)	0.006
**Marital status, N (%)** Single Married Divorced	11 (29.7) 10 (21.3) 12 (75)	26 (70.3) 37 (78.7) 4 (25)	0.001
**Accommodation, N (%)** Personal Rental	20 (27.8) 13 (46.4)	52 (72.2) 15 (53.6)	0.083
**Education, N (%)** Less than diploma Diploma & higher	18 (27.7) 15 (42.8)	47 (72.3) 20 (57.2)	0.097
**Occupation, N (%)** Unemployed Retired Housewives Employed	15 (37.5) 1 (25) 7 (38.9) 10 (26.3)	25 (62.5) 3 (75) 11 (61.1) 28 (73.7)	0.713
**Polarity of the mood episode at onset of the disorder, N (%)** Major depressive Mixed Manic	6 (30) 1 (50) 26 (33)	14 (70) 1 (50) 52 (67)	0.599
**Polarity of the mood episode at the enrollment, N (%)** Major depressive Mixed Manic	3 (42.8) 2 (66.7) 28 (31.1)	4 (57.2) 1 (33.3) 62 (68.9)	0.382
**Mean age at onset of the disorder ± SD**	23.4 ±9.2	28.3 ±9.2	0.015
**Suicidal thoughts at the time of enrollment, N (%)** Positive Negative	2 (40) 31(32.6)	3 (60) 64 (67.4)	0.536
**Suicidal attempt in first degree relative, N (%)** Positive Negative	3 (50) 30 (31.9)	3 (50) 64 (68.1)	0.309
**Axis I Co-morbidity, N (%)**			
Positive	11 (36.7)	19 (63.3)	0.628
Negative	22 (31.5)	48 (68.5)	
**Prior hospitalizations, mean ± SD**	4.2 ± 3.8	3.2 ± 2.8	0.264
**Prior hospitalizations in manic episode, mean ± SD**	4 ± 3.3	4 ± 3	0.971
**Prior hospitalizations in depressive episode, mean ± SD**	1.5 ± 1	1.4 ± 0.8	0.898
**Prior hospitalizations in unknown episode, mean ± SD **	3 ± 2.1	1.6 ± 0.9	0.36
**YMRS score at the enrollment, mean ± SD**	26.3 ± 12.3	26.6 ± 10.8	0.907
**HDRS score at the enrollment, mean ± SD**	4.1 ± 3.5	3.1 ± 2.4	0.211

**Table 2:** Multivariable logistic regression results to evaluate variables’ independent associations with suicide attempt

			**OR confidence interval (95%)**	
	**P value**	**Odds Ratio (OR)**	**Low**	**high**
**Male sex**	0.036	0.311	105	0. 0.924
**Family history of suicide attempt**	0.186	4.119	0.507	0.493
**Age at onset of the disorder**	0.035	0.937	0.882	0.995
**Education; diploma and higher**	0.886	1.082	0.369	3.177
**Divorced**	0.003	8.647	2.094	35.710

## Discussion

***A. Retrospective***

*Assessment*

The present study showed that one third of the patients had positive history of suicide attempts which is concordant with most of the reviewed studies, in which lifetime suicide attempt rates were 30-49% (9-14, 26). Bellivier et al. studied on 3684 bipolar patients and found that 30% had history of suicide attempt (11). In another study on 1098 BID patients, lifetime prevalence of suicide attempt was 35% (14).

In this study, suicide attempt had independent relationship with patients’ gender, age at onset of the disorder as well as marital status; according to multivariable logistic regression. Similarly, in several studies, female gender had significant relationship with suicide attempt (6,11,14,27). In a study on 176 Iranian BID patients by Shabani et al., female sex was correlated with higher risk of suicide attempt (27).

In some studies, early age at onset of the disease have had significant relationship with suicide attempt (11,14). In the study of Tondo et al on 2826 bipolar patients during the preceding 30 years (13), and study of Slama et al. on 307 bipolar patients (12), early age at onset of the disease have had significant relationship with suicide attempts. Shabani et al. did not find such correlation in BID patients (27).

Divorce was significantly correlated with suicide attempt. Such relationship was nearly statistically significant in another study on patients with bipolar disorder (28). Additionally, divorce could be a serious stressor for BID patients and suicide attempt rate in individuals with stressful experiences or early stressful events associated with the diseases and traumatic stresses is prominent (14, 9). In Iran, in the study of Shabani et al. correlation between marital status and suicide attempt was not observed (27).

Unlike the present study, some studies reported that depressive-onset frequency (14,28) or depression severity in previous episodes of bipolar disorder is higher in individuals with history of suicide attempts (11). This study showed no significant relationship between suicide attempt and first mood episode type or depression severity according to HDRS-7 score at the enrollment date. This contradiction could be possibly due to low percentage (20%) of first depressive episodes in our study. Nonetheless, there was also no relationship between diagnosis of the first episode and suicide attempt in other Iranian study on BID patients (27).

Our findings suggest no relationship between axis I comorbidity and suicide attempt. However, in other studies, suicide attempt has significantly emerged with anxiety disorders (27,16), alcohol use (12), alcohol and substance abuse (11) and axis I, II and III comorbidities (9) in patients with bipolar disorder. Nevertheless, there are some findings in line with the current study; for instance Vin Ryo et al. (28) found no association concerning axis I, II and III comorbidities. When it comes to substance use disorder comorbidity, it is important that what kind of substance is abused; as at least one study has substantiated no risk factor position for opioid dependence with regard to suicide attempts among BID patients (27). Taking these inconsistent and complex results into account, larger sample size may be required to reach enough statistical power to demonstrate the effect of comorbidities. Lack of relationship between suicide attempt and patients’ education, occupation and hospitalization rate in our study is consistent with Shabani et al. (27) and Vin Ryo et al. findings (28). Prior hospitalization (9,13,14) and hospitalization due to depression (12) were the factors related to suicide attempt in some other studies.

Unlike the current study, family history of suicide attempt has been significantly related to suicide attempt elsewhere (9). This finding could be affected by low percentage of suicide attempts in first degree relatives of BID patients (6%) in our study.

Apart from the factors addressed, several other factors related to suicide in patients with bipolar disorder have been presented in other studies: more depressive episodes (12,15), longer duration of the symptoms (9,14), sexual abuse (9), mania following depression (12), poor compliance (11), initial mixed episode (14), and cyclothymia(14).

***B. Prospective***

*Assessment*

In this follow-up of 100 patients for an average of 20.6 ± 12.5 months, just one patient attempted suicide (0.57% in one year; confidence interval 95%: 0.52-0.62%) which was lower than other studies.

In a 2-year follow-up study on 1556 bipolar patients, 57 cases (3.7%) attempted suicide (7). In another follow-up of 160 bipolar patients for 18 months, 20% attempted suicide (8) and a systematic review found the incidence rate of suicide attempt in bipolar patients to be 3.9% (29).

In the Systematic Treatment Enhancement Program for Bipolar Disorder (STEP-BD), 182 out of 4360 patients attempted suicide during a 5 year- follow-up (0.82% in one year) leading to death in eight of them. Almost one third of the suicide attempters, attempted more than once. Number of suicide attempts was less than expected in this study (32). Considering low sample size (N=22), the only follow-up of Iranian patients with first episode mania showed no case of suicide attempt during a mean follow-up of 17 months (31). It should be noted that the follow-up was carried out by experienced psychiatric attendants.

Variables considered as risk factors for suicide attempt according to preceding prospective studies on bipolar disorder patients include: history of previous attempts (7,8), depressive symptoms duration (7), early onset of symptoms/ disorder (8, 30), initial phase of depression (8), and hopelessness (8).

Causes of the low rate of suicide attempt in our study could be as the followings:

- Concealing suicide attempts by the patient or family members.

- Lower suicide attempts in general population in studies performed in Iran compared to the western countries (1).

- Low percentage of disease onset with depression (20%) in this study.

- Low percentage of suicidal thoughts (5%) at the time of enrollment.

- Recurrent psychiatric visits and regular follow-ups could be an important reason; as might be the case in developing no case of suicide attempt in aforementioned follow-up study on Iranian patients with first episode of mania (31). In this way, patients were intervened early as needed which could lead to better clinical course and lower rate of suicide attempt.

Some methodological limitations must be acknowledged. Firstly, our sample consisted of inpatients with BID who were not homeless, lived in a special geographical region, and had a landline and a cell phone number. Therefore, we cannot generalize the findings to other bipolar patients, homeless people, and patients living in other regions. Secondly, although patients were visited regularly and as needed, the structural assessment based on the BDPF method was only performed at months 0, 2, 6 and every 6 months afterward. So, recall bias might be significant in retrospective and insignificant in prospective evaluations.

## Conclusion

Retrospectively, results of this study reveals that BID patients, with female gender, history of divorce and lower age at onset of the disorder were at greater risk for suicide attempt; among them history of divorce had the strongest relationship. Therefore, patients with these risk factors should be carefully monitored for suicide attempt. Considering few suicide attempts in this study and some other prospective studies such as STEP-BD, it seems that development of treatment systems which actively follow the patients can effectively reduce the incidence of suicide attempts. If such systems are not available for all of these patients, patients with risk factors could be prioritized

## Authors’ Contributions

AS designed and planned the study and advised on the analysis and revised the manuscript. STE collected the data and advised on the analysis and drafted and revised the manuscript. SN and SVS helped in data collecting and revising the manuscript. MAA advised on the analysis. SK, BS, ZMB, MG, SH, SS, ZG and STA collected some of the data and helped in revising the manuscript. MT coordinated the authors and planned the study visits. 

*Financial Support*

This project was supported by a grant from Mental Health Research Center, Mental Health Research Network, and Tehran Psychiatric Institute, Iran University of Medical Sciences, Tehran, Iran.
